# Supplementary material for: The most at-risk regions in the world for high-impact heatwaves
Source: Nat Commun. 2023 Apr 25;14:2152. doi: 10.1038/s41467-023-37554-1 (PMC10130074; doi:10.1038/s41467-023-37554-1)
Supplement: Supplementary file 1 — Supplementary Information [file 41467_2023_37554_MOESM1_ESM.pdf]

The most at-risk regions in the world for high-impact heatwaves

Supplementary Figures

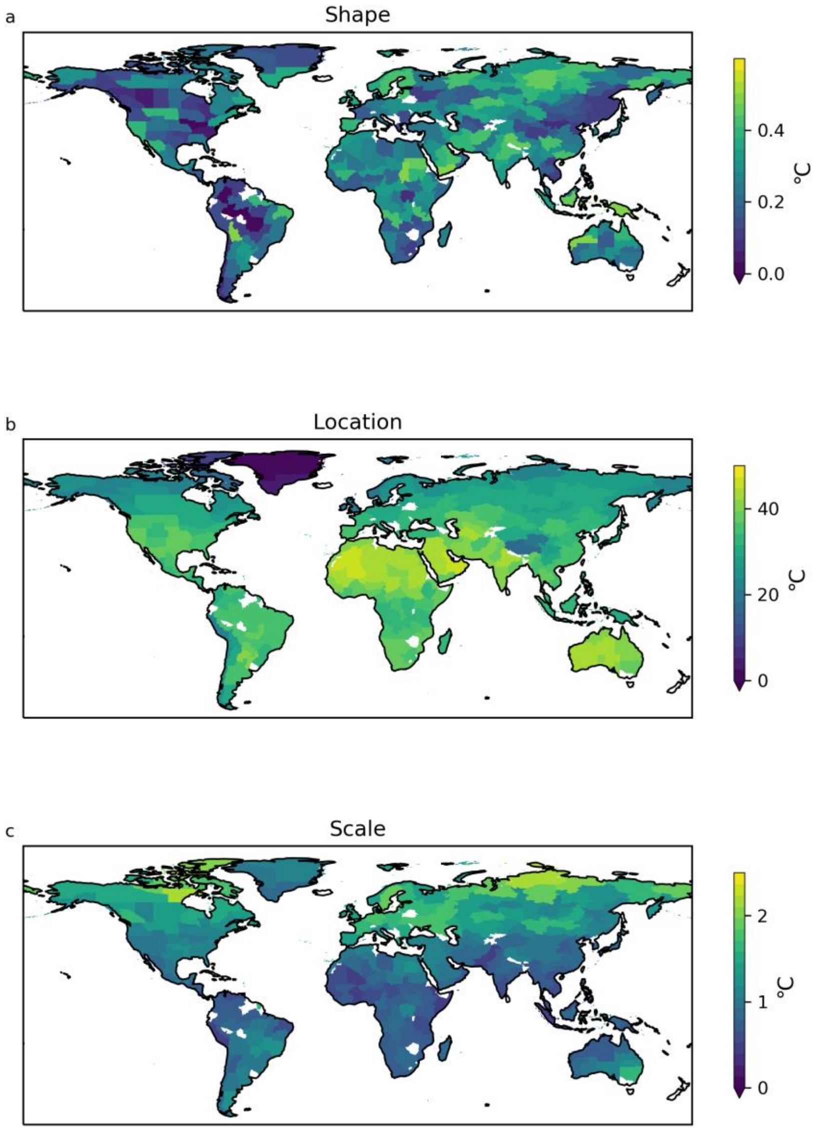

Figure S1: Generalised Extreme Value fit parameters of annual max of daily maximum temperature globally. Maps of global regions showing **a)** shape parameter, **b)** location parameter, and **c)** scale parameter. Regions taken from Stone (2019)[31].

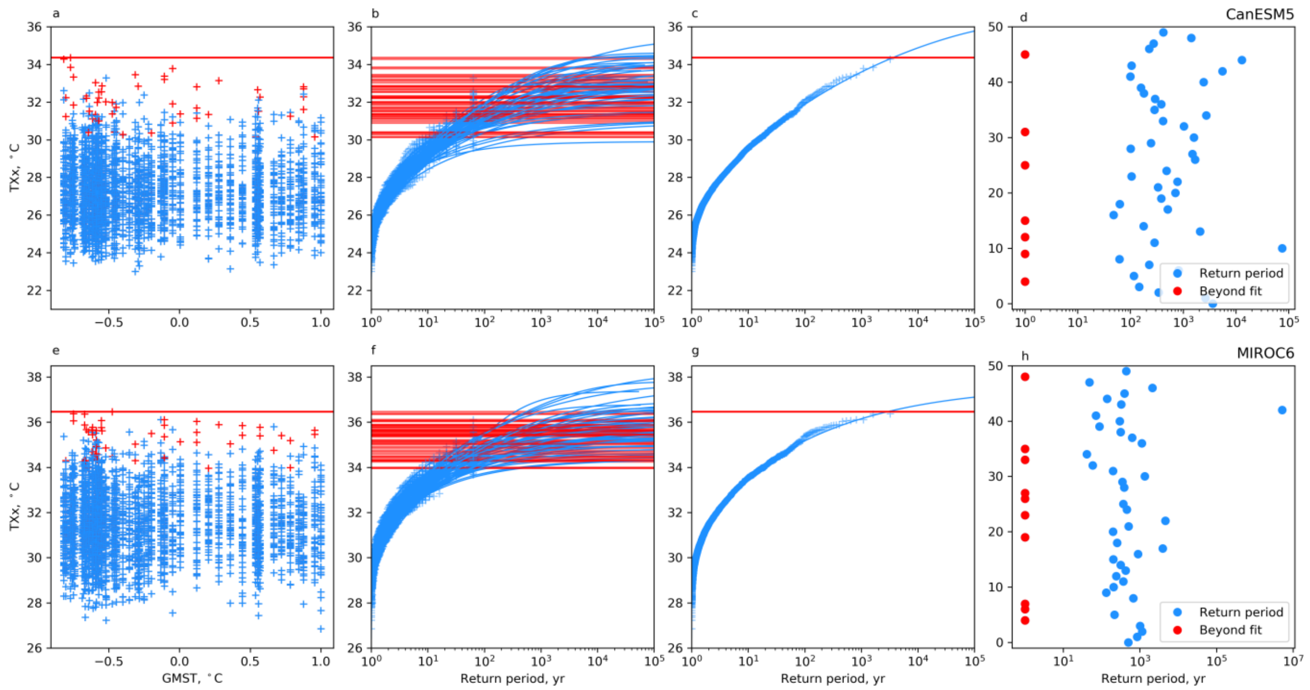

Figure S2: Generalised Extreme Value (GEV) fit of annual maximum of modelled daily maximum temperatures (*TXx*) for Alberta, Canada. a) Showing *TXx* from a 50-member ensemble of CanESM5, detrended by the CanESM5 global mean surface temperature (GMST), with each realisations maximum value in red, and the ensemble maximum marked as the red line. **b)** GEV fit of all 50 realisations, faint red lines indicate the maximum values of each realisation. **c)** GEV fit of the full ensemble, red line indicates the ensemble maximum. **d)** Return levels of the 50 realisations, with those where the record is outside the GEV fit marked in red. **e)-h)** as in a)-d) for MIROC6 ensemble.
